# Building Confidence in Psychiatric Communication and Clinical Skills: Outcomes from Psych Talk, a Multi-Session Educational Programme

**DOI:** 10.1192/bjo.2026.11277

**Published:** 2026-06-30

**Authors:** Anju Vivek Shivram, Bhavika Vajawat, Prateek Varshney, Vatsal Zapadia, Sridevi Sira-Mahalingappa

**Affiliations:** 1 South West London and St George's Mental Health NHS Trust, London, United Kingdom; 2 South London and Maudsley NHS Foundation Trust, London, United Kingdom; 3 East London NHS Foundation Trust, London, United Kingdom

## Abstract

**Aims:**

Psych Talk was developed by the British Indian Psychiatric Association (BIPA), a UK-based charitable organisation, to support doctors from diverse training backgrounds in developing effective psychiatric communication skills. The programme aimed to improve confidence in OSCE-style assessments and routine clinical encounters through structured exposure to realistic psychiatry case scenarios. Objectives included providing practical, exam-focused guidance relevant to MRCPsych examinations, strengthening rapport-building and therapeutic alliance skills, supporting the management of difficult conversations, and use of non-verbal communication.

**Methods:**

Psych Talk comprised 13 online teaching sessions delivered between June and December 2025. Sessions were held on weekends, lasted 1.5 hours each, and were chaired by UK resident doctors with support from consultant psychiatrists. Each session focused on two to three OSCE-style scenarios across psychiatric subspecialties, explored through role play, group discussion, and structured feedback. Pre-session and post-session surveys collected demographic information, career stage, confidence levels, learning sources, and qualitative feedback, with all responses analysed anonymously. Descriptive analysis evaluated changes in confidence.

**Results:**

A total of 189 participants completed the pre-session survey and 403 completed post-session feedback, with more responses obtained post-session possibly as participants completed these to receive CPD certificates. Participants represented a diverse cohort, predominantly from the UK (n=74), India (n=39), and Pakistan (n=8), with additional representation from Ireland, Egypt, the UAE, Myanmar, Malaysia, and a mixed or unspecified group (n=58). Participants who did not disclose their career stage formed the largest group (49.7%), followed by core resident doctors (35.4%). Doctors in non-training UK posts accounted for 7.4%, and IMGs outside the UK for 6.3%. Before attending Psych Talk, only around one in five participants reported feeling confident or very confident; most described themselves as somewhat confident, and 13.8% reported no confidence. After completing the sessions, nearly two-thirds reported high confidence, and those reporting no confidence fell to 1.0%. Participants valued the practical, exam-focused approach, realistic OSCE scenarios, interactive format, and clarity of guidance. Chairing by resident and consultant psychiatrists was rated excellent by nearly 75% and good by a further 20%. Mostparticipants learned about the programme through social media (37%) or colleagues (29.3%), and nearly all expressed interest in future sessions.

**Conclusion:**

Psych Talk was associated with a meaningful improvement in self-reported confidence in psychiatric communication among a globally diverse cohort. As a free and accessible charitable initiative, it highlights how shared learning environments can bridge gaps between UK and international training systems and support MRCPsych-related skill development.

